# The role of clomiphene citrate in late onset male hypogonadism

**DOI:** 10.1590/S1677-5538.IBJU.2021.0724

**Published:** 2022-02-02

**Authors:** Carlos Teodósio Da Ros, Lucas Uglione Da Ros, João Pedro Uglione Da Ros

**Affiliations:** 1 DR & G Urologistas Associados Porto Alegre RS Brasil DR & G Urologistas Associados, Porto Alegre, RS, Brasil; 2 Universidade Federal do Rio Grande do Sul Porto Alegre RS Brasil Universidade Federal do Rio Grande do Sul - UFRGS, Porto Alegre, RS, Brasil; 3 Universidade Luterana do Brasil Canoas RS Brasil Universidade Luterana do Brasil – ULBRA, Canoas, RS, Brasil

## COMMENT

The World is getting old. Aging is associated with degenerative changes in multiple organ and systems. Yet, overall, men's life expectancy increased from 63.2 years in the 1990s to 70.5 years in 2017, what represents an increase of 7.3 years in this period (1). Men's life expectancy has always been lower than women's, as several biological, ethnic and sociocultural factors weighed in, but lifestyle modifications, such as decreasing smoking and alcoholism, controlling weight and adopting physical activity, has helped to minimize this difference (2). In addition to the desire to live longer, people value more and more quality of life, and among health issues that involve quality of life, we have what concerns cognition, reasoning, libido and the feeling of well-being. These aspects are all tightly related to testosterone, which is the main male hormone, responsible for all of this and, still, for strength and muscle mass, maintenance of bone structure, penile erections, etc. Greater attention has been given to health in this group of older male patients, not least because there is a high prevalence of metabolic and psychological alterations related to aging among them (3).

Symptomatic late-onset hypogonadism (LOH) is a clinical and laboratory syndrome that accompanies male aging and is associated with hormonal profile changes, which negatively affect libido, sexual function, mood, behavior, lean body mass, and bone density, that is, it affects not only the homeostasis of the organism but also its psychological function (3–5). Currently, the most common treatment of symptomatic LOH is testosterone therapy with various options of administration: transcutaneous, buccal, oral or intramuscular. Once the indications are observed, i.e., a clinical picture associated with laboratory confirmation, there are safe and efficient replacement alternatives (6, 7). In recent years, the prescription of testosterone has increased greatly, however men who wish to maintain their reproductive potential are not completely warned of the risks of using exogenous testosterone (8). But as life expectancy is increasing, it is perceived that the first marriage occurs later in life, consequently the intention of having the first child is postponed, so men also tend to be parents later nowadays. If a man who desires later paternity is hypogonadal, testosterone replacement can bring an unwanted damage to spermatogenesis (8, 9).

Testosterone replacement has been used as a treatment for symptomatic hypogonadal men and the administration of exogenous testosterone is the pillar of this therapy. The goal of replacement is to maintain physiological hormone levels. But there are some contraindications to its use, such as in those individuals who still desire offspring. So, we need alternatives to raise testosterone levels in these cases, without providing replacement and rather stimulate endogenous production of the hormone, and among them is clomiphene citrate, the theme of this current review.

### Late onset male hypogonadism

**Figure 1:**
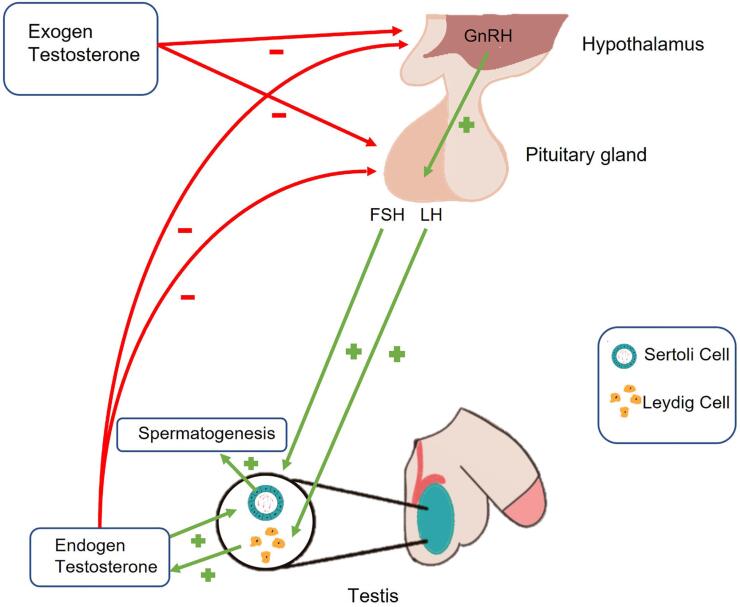
Sites where endogenous and exogenous testosterone can act on the hypothalamic pituitary gonadal axis.

Testosterone production is controlled by the hypothalamic-pituitary-testicular axis, where the gonadotropin-releasing hypothalamic hormone (GnRH) stimulates the pituitary gland to produce gonadotropins (luteinizing hormone (LH) and follicle stimulating hormone (FSH)). LH acts on testicular Leydig cells, stimulating testosterone production, while FSH, along with testosterone, stimulates spermatogenesis (8) (Figure-1). Unlike women, men do not show a cessation of hormone production, but rather a gradual decrease of 40 years of age. This leads to a deficiency of androgens (10–12), which can compromise both the quality of life and the functioning of certain organs (3–5). It is already well established that this decrease in testosterone (TT) can cause sarcopenia, muscle weakness, increased adipose tissue, fatigue, lack of motivation, depression, irritability, anemia, lower memory, and reasoning capacity. This late-onset hypogonadism is also related to diabetes mellitus, metabolic syndrome, coronary artery disease, and cardiovascular disease in general. In addition to the reduced serum TT levels and the signs and symptoms listed above, individuals report decreased in libido, less erotic thoughts, fewer nocturnal and morning erections, and they also present erectile dysfunction (3).

The decrease in total testosterone occurs in a rate of 1% per year and free testosterone, 2% per year, from the age of 40. This decrease is due to a smaller number and minor function of Leydig cells, changes in the hypothalamus-pituitary-gonadal axis and increased sex hormone binding globulin (SHBG) (3, 4, 10). Hypogonadism in aging is also associated with increased body weight and adipose tissue, resulting from peripheral conversion of testosterone into estradiol, and the negative feedback from estradiol in the pituitary gland results in a low LH secretion despite a low testosterone level. Clinical syndrome is directly related to some risk factors including metabolic syndrome, diabetes mellitus, sleep apnea and chronic obstructive pulmonary disease, rheumatoid arthritis, hemochromatosis and other chronic diseases, and also is related to a higher risk of death in general (3, 8).

The prevalence of hypogonadism is not yet completely determined because there are still no longitudinal studies with large enough casuistry to obtain this data. Morley et al., in the Study of Baltimore (13), found a higher prevalence of hypogonadism in patients over 50 years of age. The literature shows a prevalence of hypogonadism ranging from 2 to 38% of the adult male population (5, 14).

### Testosterone Replacement Therapy

Once hypogonadism (clinical and biochemical) has been diagnosed, hormone replacement should be initiated, provided that there are no contraindications. The treatment for hypogonadism most used is the administration of exogenous testosterone, which can be administered in oral, buccal, intramuscular or transdermal form (gel or patch) (4, 10).

In symptomatic patients, recovery of sexual desire is one of the first responses of TT replacement (4). Testosterone also exerts an important effect on body composition, increasing lean mass and decreasing fat mass (4, 10). In the wake of the recovery of physiological hormone levels, we can see response in the most different areas: decrease in negative thoughts, improvement in bone mineral density, increases in lean mass and in muscle strength (4, 15, 16). Overall, the recovery of normal hormone levels improves the quality of life of patients (3, 11).

Testosterone replacement can cause suppression of the hypothalamic-pituitary-gonadal axis via negative feedback mechanism and the clinical manifestations of the axis malfunction comprise reduction of both testicular size and sperm count. So, patients who still have an interest in offspring, should not receive any form of exogenous testosterone (3, 10, 12).

When the use of exogenous testosterone is contraindicated, the patient has an interest in offspring, or there are side effects related to gel or intramuscular applications, we can use dopaminergic agonists, gonadotropins, aromatase inhibitors or selective inhibitors of androgenic receptors (SERM), which are all effective in treating hypogonadism (12).

Exogenous gonadotropins (human chorionic gonadotropin - hCG, human menopausal gonadotropin - hMG, highly purified FSH - hpFSH, human recombinant FSH - rhFSH, long-lasting analogue FSH - alpha coryfolitropin) can be used to replace endogenous testosterone production by stimulation of Leydig cells due to their similarity with LH, and their use is quite common in cases of oligospermia or even azoospermia (17). There are several treatment regimens, and these drugs can be used intramuscularly or subcutaneously. Data are limited in the treatment of late-onset hypogonadism, but it is a feasible option that preserves fertility because it does not suppress the hypothalamus-pituitary-gonadal axis, however it has an elevated cost (8, 12).

Aromatase inhibitors (anastrozol and letrozole) inhibit the conversion of androgens into estrogens, preserving TT levels and limiting estrogen production. By doing so, they will prevent negative estradiol feedback on the production and release of gonadotrophins at the hypothalamic level. As a result, there will be a greater stimulation towards testosterone production (8, 11, 12). Unlike selective estrogen receptor modulators (SERM), aromatase inhibitors reduce estrogen levels (12). Its use in male infertility, to stimulate spermatogenesis, and in hypogonadism, to increase TT, is off label, like any other estrogen modulator. The dose of anastrozol usually used is 1mg 1x/day (8). Studies show increased testosterone levels, but the analysis of clinical response related to sexuality, body composition or muscle strength is still small. In addition, there is an association with a lower bone mineral density in patients when compared to testosterone replacement, since estrogens participate in various physiological functions, including bone metabolism, cardiovascular health, spermatogenesis, and cognition (4, 12).

### Clomiphene citrate (CC)

Clomiphene Citrate (CC) is a weak selective estrogen receptor modulator (SERM) antagonist at the hypothalamus level. It attaches to the receiver for an extended period, reducing the availability of these receptors. As estradiol exerts negative feedback on the hypothalamus, down-regulating the production and the release of gonadotropic-releasing hormone (GnRH), CC will increase hormone levels and consequently increase the stimulus on the pituitary gland. CC has the ability to compete with estradiol for the estrogen receptors at the level of the hypothalamus, so this drug blocks the normal negative feedback of circulating estradiol on the hypothalamus, preventing estrogen from lowering the output of GnRH. During clomiphene therapy, the frequency and amplitude of GnRH pulses increase, stimulating the pituitary gland to release more FSH and LH. Consequently, sperm and testicular testosterone productions are stimulated (18, 19).

This drug was developed for the treatment of female infertility in the 1960s, but it has also been used for treating male hypogonadism and infertility since then (20). The usual dose is 25-50mg per day, and testosterone elevation takes place after 4 weeks of treatment (12, 21). CC is effective in increasing TT levels as well as improving symptomatology caused by hypogonadism (22). But unlike the testosterone formulations employed to correct hormone levels, this drug preserves the patients’ fertility. However, it is not effective in those cases where LH and FSH are elevated, characterizing primary hypogonadism (8, 12).

Tenover et al. used CC in 5 young patients (between 26 and 33 years) and 5 over 65 years of age, at a dose of 100mg/day for 8 weeks. They noticed that testosterone levels rose in both groups, more significantly in the youth group. These findings show that the alternative is effective regardless of age group (21).

Katz et al. used CC to treat hypogonadism of 86 young and infertile patients (mean age of 29 years) for an average period of 19 months. The dose used ranged from 25 to 50mg every other day and the aim of the study was to reach a testosterone value of approximately 550ng/dL. This was achieved in all patients and an important improvement in libido, in the feeling of well-being, the mood and in physical performance were also observed (22). No side effects were observed during the study. The same authors observed a group of 76 hypogonadal patients (testosterone levels <300ng/dL), with a mean age of 46 years, who were treated with CC. The treatment of all of these patients was successful, but they realized that those who had normal volume testicles (>14mL) and those with LH levels lower than 6 IU/mL had a better response to treatment (23).

Guay et al. observed an increase in testosterone and an improvement in erections in 173 hypogonadal men who complained of erectile dysfunction. Patients used CC 50mg 3x/week for 4 months (24).

In a comparison between exogenous testosterone and CC, a group of 52 patients received injections or TT gel, while 23 patients were instructed to use CC. The authors observed a similar increase in TT levels in both treated groups, as well as improvement of clinical parameters (25).

Taylor and Levine found elevation of TT levels in 104 patients treated with testosterone gel or CC. In this study, 65 of the patients used CC 50mg 2/2 days and were followed for up to 23 months. TT increased from mean pre-treatment value of 277ng/dL to a mean value of 573ng/dL after. The result showed that CC serves as an alternative to the use of exogenous testosterone (gel), with few side effects and a much lower cost (15).

In a group of 36 young hypogonadal patients (mean age 39 years), Shabsigh et al. observed an important increase in TT levels (247.6ng/dL, mean pre-treatment; 610ng/dL, mean post-treatment) after the use of CC at a dose of 25mg/day for 4 to 6 weeks (26).

In a comparison between CC (25mg/day) and anastrozol (1mg/day), Helo et al. treated 26 hypogonadal and infertile men for 12 weeks, observed an increase in TT levels in both groups, but more significant with CC (27).

In a study involving young patients (mean age of 36.5 years), who were both obese and hypogonadal, the use of CC 50mg/day for 12 weeks brought benefit on sexual function, lean mass and muscle mass, demonstrating improvement in the hormonal profile of patients, as well as body composition (28). Lim and Fang also observed improved in libido, in the erections and in the feeling of well-being in a group of 5 hypogonadal patients with chronic renal failure who received CC 100mg/day for a period of up to 12 months. They also showed that these patients remained eugonadic for up to 5 months after discontinuation of treatment (29). On the other hand, Marconi et al. used CC 50mg/day for 50 days in 27 patients with hypogonadism; there was a significant increase in hormone levels, which decreased again after discontinuation of the drug (30). Hormone replacement, in general, is of continuous use. The use of testosterone replacement is uninterrupted, because when it is suspended, the patient becomes hypogonadic again, and experiences the recurrence of the clinical picture that motivated the initial consultation. This is probably the case for the therapeutic alternatives to hormone replacement, such as CC, which should also be continuous as was showed by this study by Marconi et al. (30).

There are also long-term publications, such as the study by Moskovic et al. who treated 46 hypogonadal patients with CC and had a follow-up period of up to 3 years. Patients had a mean age of 44 years in the baseline and used 25 to 50mg of CC every other day. In the end of follow-up, a TT elevation was shown, as expected (228ng/dL in baseline, 582ng/dL in the third year of treatment) and virtually no side effects occurred (31).

Da Ros and Averbeck (32) observed an improvement in TT levels and in libido in a group of 125 hypogonadal patients, with a mean age of 62 years, who were treated with 25mg/day of CC for 3 months. There was an average increase in TT levels from 309ng/dL to 642ng/dL. And they had virtually no notable side effects.

Studies show good tolerability and only a few mild side effects, such as hot flashes, headache, gynecomastia, dizziness and fatigue (11, 20, 25). CC is a good alternative for the treatment of symptomatic hypogonadal patients and has as advantages the absence of hypothalamus-pituitary-testicular axis block, the fact that it does not suppress spermatogenesis and does not cause polycythemia and its low cost (18, 31, 32).

## CONCLUSION

In our modern World, the desire for later paternity has been frequent, mainly due to socio-economic-cultural issues, which include longer life expectancy, greater use of contraceptives and the large female presence in the labor market. Men are thus, more frequently experiencing fatherhood in late adulthood. The average age of paternity increased, in the last 40 years, from 27.4 to 30.9 years, and in the 1970s, 6.1% of men became a father over 40 years; between 2011 and 2015, this percentage rose to 12.7% (9). However, the late pregnancy, besides being more difficult due to changes in the hypothalamus-pituitary-gonadal axis (hypogonadism) and spermatogenesis, is related to harmful DNA damage accompanied by congenital mutations, autism and schizophrenia (33). Despite the risks, the trend of later paternity shows us the importance of maintaining good health and also of tending to the men's fertility.

Along with the well-established non-pharmacological interventions, such as lifestyle changes, that can be made in this population, testosterone replacement remains the standard treatment for those men with androgenic deficiency of male aging. It is not an option for those who desire offspring, since testosterone replacement decreases sperm production, or for those who have a difficult-to-control polycythemia. For these hypogonadal patients, there are other alternatives such as CC.
